# Myeloid sarcoma of the oral cavity: A case report and review of 89 cases from the literature

**DOI:** 10.4317/jced.53935

**Published:** 2017-09-01

**Authors:** Bruno-Augusto-Benevenuto de Andrade, Renan-de Barros Farneze, Michelle Agostini, Ellen-Brilhante Cortezzi, Aline-Corrêa Abrahão, Marcia-Grillo Cabral, Alicia Rumayor, Mário-José Romañach

**Affiliations:** 1 DDS, PhD, Oral Pathology, Department of Oral Diagnosis and Pathology, School of Dentistry, Federal University of Rio de Janeiro (UFRJ), Rio de Janeiro, Brazil; 2 DDS, MSc, Oral Pathology, Department of Oral Diagnosis and Pathology, School of Dentistry, Federal University of Rio de Janeiro (UFRJ), Rio de Janeiro, Brazil; 3 DDS, PhD , Oral Medicine, Department of Oral Diagnosis and Pathology, School of Dentistry, Federal University of Rio de Janeiro (UFRJ), Rio de Janeiro, Brazil; 4 DDS, PhD, Oral Pathology, Department of Oral Diagnosis, Piracicaba Dental School, University of Campinas (FOP-UNICAMP), Piracicaba, Brazil

## Abstract

Myeloid sarcoma is a tumor mass of immature myeloid or granulocytic cells that affects extramedullary anatomic sites, including uncommonly the oral cavity. A 24-year-old female was referred for evaluation of a fast growing painful gingival swelling lasting 2 weeks, associated with fever, fatigue, and cervical lymphadenopathy. Intraoral examination showed a bluish swelling on the right posterior lower gingiva exhibiting necrotic surface. Incisional biopsy of the gingival lesion displayed diffuse infiltration of undifferentiated tumor cells with granulocytic appearance, strongly immunopositive for CD99, myeloperoxidase and Ki-67 (60%), and negative for CD20, CD3, CD34 and TdT. Blood tests presented a severe pancytopenia, and genetic analysis confirmed the diagnosis of acute promyelocytic leukemia. The final diagnosis was of oral myeloid sarcoma associated with acute promyelocytic leukemia with t(15;17). The patient was submitted to chemotherapy but died of the disease one month later. The clinicopathologic and immunohistochemical features of the present case are compared with the 89 cases of oral myeloid sarcoma previously reported in the English-language literature.

** Key words:**Myeloid sarcoma, chloroma, granulocytic sarcoma, gingiva, oral, acute promyelocytic leukemia, acute myeloid leukemia.

## Introduction

Myeloid sarcoma (MS), also known as granulocytic sarcoma or chloroma, is a tumor mass of immature myeloid cells that usually occurs in an extramedullary site or bone of male patients in the sixth decade of life (1). MS has been associated with acute myeloid leukemias (AML) or other myeloproliferative disorders (2-3). Treatment and prognosis of MS depends on the hematological status and clinical presentation (4).

The microscopical features of MS include the presence of immature myeloblasts within a dense inflammatory background, which are better identified after careful histological and immunohistochemical evaluation (4-5). Some markers are useful to confirm an immature myeloid phenotype of tumor cells, such as myeloperoxidase (MPO), CD68, CD117, CD34, and CD99 (6).

Oral involvement by MS is uncommon. To the best of our knowledge, only 89 cases of oral MS have been published in the English-language literature so far, and only four of them were associated with acute promyelocytic leukemia (1-15). Herein, we report an additional case of oral MS in a 24-year-old female with acute promyelocytic leukemia, including a review of the literature.

## Case Report

**Figure 1:**
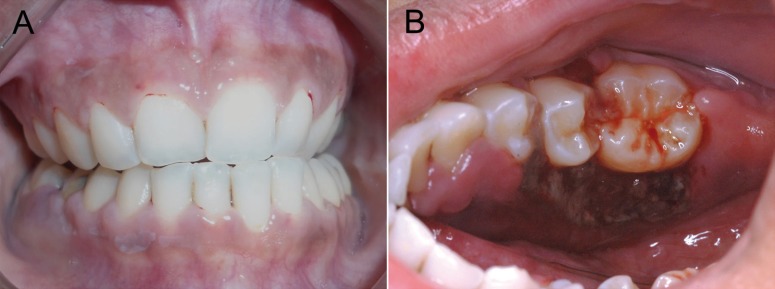
Clinical features of oral myeloid sarcoma. (A) Intraoral examination showing pale oral mucosa, blood accumulation within the gingival sulcus of various teeth, and a normal colored swelling on the buccal posterior lower gingiva of the right side. (B) Brownish swelling with ulceration on the lingual aspect of the right posterior lower gingiva exhibiting also necrotic and bleeding surface.

**Figure 2:**
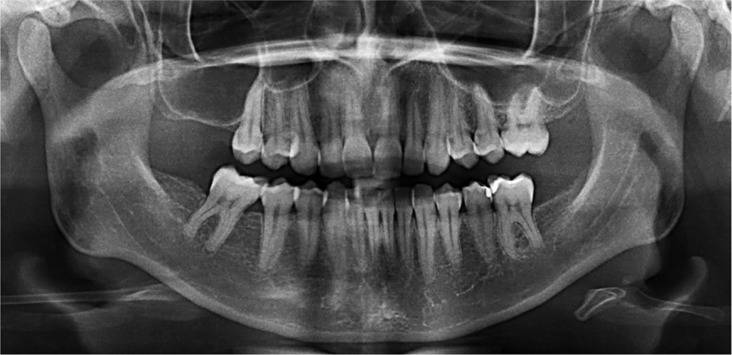
Panoramic radiography exhibiting absence of bone involvement.

A 24-year-old female was referred by a general dentist for evaluation of a fast growing gingival swelling that had been present for 2 weeks. The patient reported a 3-weeks history of fever and fatigue. Physical examination revealed cervical lymphadenopathy, and intraoral examination showed discrete areas of clotted blood within the gingival sulcus of some teeth, and a 3 cm painful brownish swelling with necrotic and bleeding surface localized in the right posterior lower gingiva (Fig. 1). Radiographic examination of the mandible showed no bone involvement (Fig. 2). Under the presumptive clinical diagnosis of lymphoma/leukemia, a blood study was requested and the patient was submitted to an incisional biopsy.

**Figure 3:**
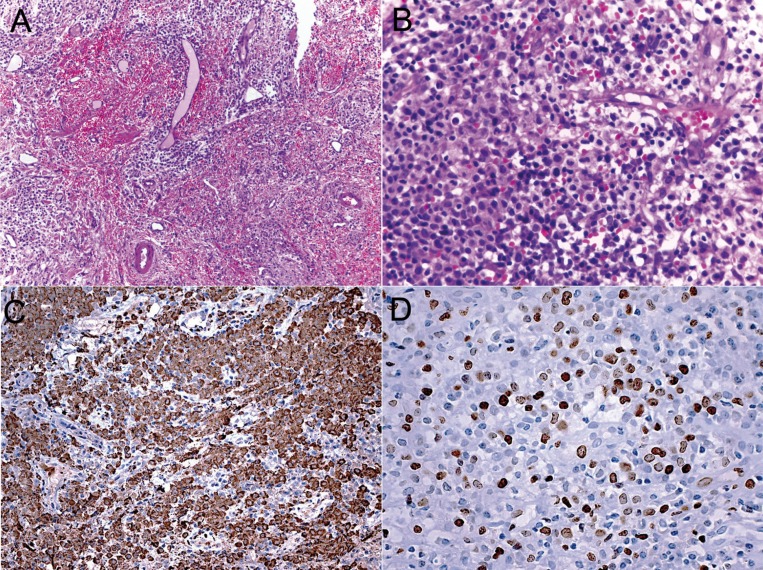
Histopathological and immunohistochemical features of oral myeloid sarcoma. (A) Diffuse infiltration of the gingival connective tissue by sheets of poorly differentiated hematopoietic cells, exhibiting dense nuclei, and basophilic cytoplasm within a background of capillary proliferation and abundant erythrocyte extravasation (HE, 100X). (B) The infiltrate is composed mostly of myelocytes promyelocytes, and myeloblasts. The cells are large in size and round to oval in shape, and the cytoplasm was mild to moderately basophilic (HE, 400X). Tumor cells showed a strong positivity for (C) myeloperoxidase, and (D) Ki-67 (Immunoperoxidase, 400X).

The gingival specimen showed a diffuse connective tissue infiltration by poorly differentiated blast-like cells intermingled with chronic inflammatory infiltrate. Tumor cells were large, round to oval, with mild to moderately basophilic cytoplasm containing granules, and round to folded nuclei with fine chromatin. Occasional mitotic figures were found (Fig. 3). By immunohistochemis-try, tumor cells were intensely positive for myeloperoxidase (dilution 1:5000, polyclonal, Dako, Carpinteria, CA, USA) and CD99 (dilution 1:100, clone 12e7, Dako, Carpinteria, CA, USA), and negative for CD20 (dilution 1:1000, clone L26, Dako, Carpinteria, CA, USA), CD3 (dilution 1:500, polyclonal, Dako, Carpinteria, CA, USA), CD34 (dilution 1:50, clone QBEnd10, Dako, Carpinteria, CA, USA), and TdT (dilution 1:50, polyclonal, Dako, Carpinteria, CA, USA). Ki-67 (dilution 1:100, clone MIB-1, Dako, Carpinteria, CA, USA) labeling was high, with 60% of tumor cells positive (Fig. 3). Blood findings showed pancytopenia (0.7 x109/L leucocytes, 31 x 109/L platelets, hemoglobin 6.3 g/dl, and hematocrit 18.6%) and the specific chromosomal translocation t(15;17) revealed by genetic analysis confirmed the diagnosis of acute promyelocytic leukemia with recurrent genetic abnormality. The final diagnosis of the oral lesion was myeloid sarcoma associated with acute promyelocytic leukemia with t(15;17). The patient was then referred to a hematology-oncology service, and submitted to chemotherapy including all trans retinoic acid (ATRA), idarubicin, and cytarabine. Unfortunately, the patient died one month later after severe hemorrhagic episodes.

## Discussion

Myeloid sarcoma (MS) is an extramedullary solid tumor composed of myeloblasts or immature cells of granulocytic lineage and erythroid precursors (2,3). First described by Burns and King in 1811, MS is also referred as “chloroma” due its green colored appearance when exposed to air, by the presence of myeloperoxidase in the tumor cells (1). Since the greenish color is not a consistent clinical finding and considering a proved granulocytic origin, the terms granulocytic sarcoma or myeloid sarcoma are preferably adopted by most authors (1-3).

MS is mainly found in the bone and soft tissues, but it may affect virtually any site of the body such as the skin, lymph nodes, orbit and eye, oral cavity, bronchi, pericardium, peritoneum, gastrointestinal tract, kidney, reproductive organs, breast, and bladder (1-4). Considering the 89 cases of oral MS previously published in the English literature, the average age of the patients is 45 years (ranging from 1 to 89 years), with slight predilection for females (1,2:1). The mandible is the most common affected site (28 cases), followed by maxilla (20 cases), gingiva (13 cases), and palate (seven cases) (1-15). MS affected multiple intraoral anatomical sites in eight cases, six of them with concomitant involvement of maxilla and mandible (5,7,8,11,13-15). The most common clinical feature of oral MS is a painful swelling or nodule with reddish to brownish-colored ulcerated surface. From all cases reviewed from the literature, only five exhibited evident greenish coloration (1,2,4,10,12). The clinical differential diagnosis is wide, ranging from lymphoma, squamous cell carcinoma and sarcomas to benign reactive or inflammatory lesions. The diagnosis of oral MS is usually based on histopathological and immunohistochemical analysis, and a history of symptoms associated with hematological diseases that might be absent (7-9). In the present case, the patient was a 24-years-old female patient, who was diagnosed concomitantly with a subtype of acute myeloid leukemia. The clinical appearance of the present case was consistent with a malignant tumor, showing an ulcerated and painful brownish-colored swelling in the lingual aspect of the lower posterior gingiva with no bone involvement. The identification of pale-appearing mucosae and areas of coagulated blood within the gingival sulcus led us to suspect of a leukemia, which was confirmed after blood test and genetic analyses.

Microscopically, oral MS exhibits variable numbers of primitive, poorly differentiated cells with granular cytoplasm, round to oval nuclei with well-defined membrane and prominent nucleoli, intermingled with reactive inflammatory infiltrate. The tumor cells show different stages of myeloid differentiation, including the eosinophilic myelocytes and blastic cells with minimal granulocytic differentiation (9). The microscopical differential diagnosis of oral MS includes diffuse large B-cell lymphoma, Burkitt lymphoma, lymphoblastic lymphoma, and poorly-differentiated squamous cell carcinoma (7). The histopathological diagnosis can be difficult, especially in cases of prominent reactive inflammatory infiltrate background and limited correlation with clinical features. Immunohistochemical analysis is necessary to prove the granulocytic origin of tumor cells (6), which are usually positive for myeloperoxidase (MPO) and CD99, important markers for the diagnosis of myeloid sarcoma (9). In the present case, the identification of the granulocytic appearance of the tumor cells was difficult on hematoxylin and eosin-stained slides, and the immunohistochemical positivity for myeloperoxidase in a cytoplasmic pattern and for CD99 in a membrane pattern was essential to achieve the final diagnosis.

MS may precede, coexist with, or follow a presentation of acute myeloid leukemia (AML), but might also result from an acute blastic transformation of myelodysplastic syndromes or myeloproliferative neoplasms (6). In fact, the diagnosis of MS is currently considered synonym of AML, and the same chemotherapeutic regimens are used (6). Acute promyelocytic leukaemia (APL; also previously known as AML-M3) accounts for around 8% of all AML cases, occurring mainly in early adulthood. APL is characterized by the predominance of abnormal promyelocytes in the bone marrow and identification of a specific chromosomal translocation t(15;17)(q24.1;q21.1) resulting in a fusion transcript between the genes promyelocytic (PML on chromosome 15) and retinoic acid receptor alpha (RARA on chromosome 17). The most common diseases associated with oral MS are AML (43 cases), followed by myelodysplastic syndrome (11 cases), and chronic myeloid leukemia (seven cases), nevertheless 14 patients with oral MS did not present nor developed associated leukemia or myelodysplastic neoplasms (1-15).

Approximately 90% of patients with APL have shown complete remission of disease after the advent of ATRA and anthracycline-based therapeutic regimens (7). However, the rate of early induction death is still high in patients with APL, and one of the most common causes is hemorrhage, as observed in the present case. Only four out of 89 cases of oral MS reported in the English literature were associated with APL (3), and one died of the disease, as the present case. From all oral MS cases reported in the literature, 53 patients died of disease, 16 had no evidence of disease, and 11 were alive with disease (1-15).

In summary, oral MS is uncommon, with clinical and microscopical features that may mimic inflammatory lesions or other malignant tumors. Careful morphological and immunohistochemical analyses, correlating with clinical data are necessary to establish the diagnosis of oral MS. Clinicians and oral pathologists should consider MS when evaluating gingival swellings and ulcerations in patients with clinical findings suggestive of hematological abnormalities.
